# Chronic visceral acid sphingomyelinase deficiency (Niemann-Pick disease type B) in 16 Polish patients: long-term follow-up

**DOI:** 10.1186/s13023-019-1029-1

**Published:** 2019-02-22

**Authors:** Patryk Lipiński, Ladislav Kuchar, Ekaterina Y. Zakharova, Galina V. Baydakova, Agnieszka Ługowska, Anna Tylki-Szymańska

**Affiliations:** 1 Department of Pediatrics, Nutrition and Metabolic Diseases, The Children’s Memorial Health Institute, Warsaw, Poland; 2 Institute of Inherited Metabolic Disorders, First Faculty of Medicine, Charles University and General University Hospital, Prague, Czech Republic; 3 Department of Inherited Metabolic Diseases, Research Center for Medical Genetics, Moscow, Russian Federation; 4 Department of Genetics, Institute of Psychiatry and Neurology, Warsaw, Poland

**Keywords:** Acid sphingomyelinase, Chronic visceral acid sphingomyelinase deficiency, Hepatosplenomegaly, Chitotriosidase, Lysosphingomyelin, Lysosphingomyelin-509

## Abstract

**Background:**

Acid sphingomyelinase deficiency (ASMD), due to mutations in the sphingomyelin phosphodiesterase 1 (*SMPD1*) gene, is divided into infantile neurovisceral ASMD (Niemann-Pick type A), chronic neurovisceral ASMD (intermediate form, Niemann-Pick type A/B) and chronic visceral ASMD (Niemann-Pick type B).

We conducted a long-term observational, single-center study including 16 patients with chronic visceral ASMD.

**Results:**

12 patients were diagnosed in childhood and 4 others in adulthood, the oldest at the age of 50. The mean time of follow-up was approximately 10 years (range: 6 months – 36 years). Splenomegaly was noted in all patients at diagnosis. Hepatomegaly was observed in 88% of patients. Moderately elevated (several-fold above the upper limit of normal values) serum transaminases were noted in 38% of patients. Cherry-red spots were found in five Gypsy children from one family and also in one adult Polish patient, a heterozygote for p.delR610 mutation. Dyslipidemia was noted in 50% of patients. Interstitial lung disease was diagnosed in 44% of patients. Plasmatic lysosphingomyelin (SPC) was elevated in all the patients except one with p.V36A homozygosity and a very mild phenotype also presenting with elevated plasmatic SPC-509 but normal chitotriosidase activity. The most common variant of *SMPD1* gene was p.G166R. We found a previously unreported variant in exon 2 (c.491G > T, p.G164 V) in one patient.

**Conclusions:**

Chronic visceral ASMD could constitute a slowly progressing disease with a relatively good outcome. The combined measurement of lysosphingomyelin (SPC) and lysospingomyelin-509 (SPC-509) is an essential method for the assessment of ASMD course.

## Introduction

Acid sphingomyelinase deficiency (ASMD), due to mutations in the sphingomyelin phosphodiesterase 1 (*SMPD1*) gene, was historically divided into two phenotypes: Niemann-Pick disease type A (OMIM 257200) – a neuronopathic, rapidly progressing and fatal disorder, and Niemann-Pick disease type B (OMIM 607616) – a non-neuronopathic, slowly progressive, visceral disorder [1, 2]. An intermediate neurologic phenotype, Niemann-Pick disease type A/B, was also reported in the literature [3, 4].

Recently, novel terminology for ASMD phenotypes was proposed including the following: infantile neurovisceral ASMD (Niemann-Pick type A), chronic neurovisceral ASMD (intermediate form, Niemann-Pick type A/B) and chronic visceral ASMD (Niemann-Pick type B) [5].

So far, only few longitudinal observational studies on ASMD have been published. However, the natural history of chronic visceral ASMD has not been exhaustively described yet due to a broad clinical heterogeneity and the lack of long-term follow-up of the patients [6–9].

The aim of this study was to present long-term follow-up of chronic visceral ASMD patients concerning the clinical, biochemical and molecular findings. The usefulness of plasma biomarkers: lysosphingomyelin (SPC) and lysosphingomyelin-509 (SPC-509) was also presented.

## Patients and methods

### Patients

The article presents a long-term observational, single-center study of patients with chronic visceral ASMD. 16 patients who were diagnosed and followed-up at the Children’s Memorial Health Institute (Warsaw, Poland) were enrolled into the study.

### Methods

The chart review of patients’ medical records concerning the demographics, the first presented signs and symptoms, age at diagnosis, as well as biochemical (acid sphingomyelinase activity in leukocytes, cultured skin fibroblasts or dried blood spot, serum chitotriosidase activity, lysosphingomyelin (SPC) and lysosphingomyelin-509 (SPC-509) levels in plasma, and also aspartate (AST) and alanine (ALT) aminotransferases, platelet count (PLT), total serum cholesterol (TC), high-density lipoprotein-cholesterol (HDL-C), low-density lipoprotein-cholesterol (LDL-C), triglycerides (TG), and molecular data (S*MPD1* gene mutations) were collected. Data on pulmonary function included spirometry and imaging of the lungs (X-ray or CT-scan). Hepatosplenomegaly was assessed with imaging (ultrasound or CT-scan).

Ethical approval was obtained from the Children’s Memorial Health Institute Bioethical Committee, Warsaw, Poland.

### Laboratory analyses

The diagnosis of ASMD was confirmed by the demonstration of reduced ASM activity in peripheral blood leukocytes or cultured skin fibroblasts. In one patient, only a dried blood spot was available. ASM activity in leukocytes was measured with the 2-N-hexadecanoylamino-4-nitrophenylphosphorylcholine as a substrate [10]. As regards patients in whom ASM activity in leukocytes was inconclusive the second measurement in cultured skin fibroblasts was performed. Chitotriosidase activity was measured in plasma samples with a spectrofluorometric method as presented by Hollak et al. [11]. In cases suggestive of chitotriosidase deficiency, screening for the 24 bp-duplication was performed. The methodological details of plasmatic SPC and SPC-509 quantitation were characterized previously in a paper by Kuchar et al. [12]. Sequence analysis of the *SMPD1* gene was performed either by targeted analysis of common mutations or whole gene sequencing. All identified molecular variants were prioritized according to the predicted effect on the protein.

Individual patients’ characteristics are summarized in Table 1.

**Table 1:** Individual chronic visceral ASMD patients' characteristics

Pt	Sex Ancestry	First signs and symptoms + age	Age at diagnosis	^*^Acid sphingomyelinase activity in leukocytes (L), skin fibroblasts (F) or dried blood spot (B); % of residual activity	^**^Chitotriosidase activity (age at measurement)	^***^SPC and SPC-509 measurement in plasma; Age at measurement	Molecular variant/protein effect	Natural history – the last follow-up data	Outcome, current age
1	M Gypsy	6 mo mild HS, cholestatic jaundice (TB 6.5, DB 3.0), elevated ST (AST 112, ALT 39), coagulopathy (INR 1.8), lipid serum profile – nk	1 y	3.9 nmol/mg protein/18 h (L); 5.6%	40 (1 y)	n.a.	c.1177 T > G, p.Trp393Gly/ c.1177 T > G, p.Trp393Gly	mild HS, thrombocytopenia (PLT 14), elevated ST (AST 160, ALT 60), cholestasis (TB 10.6, DB 6.5), coagulopathy (INR 2.8), hypoalbuminemia, ascites, interstitial lung disease on chest X-ray and CT, lipid serum profile – nk, cherry red spot, death due to *Neisseria meningitidis* sepsis, in liver biopsy post-mortem cirrhosis hepatis	Died at 1.5 y
2	F Gypsy	7 mo mild HS, cholestatic jaundice (TB 4.9, DB 3.5), elevated ST (AST 200, ALT 80), coagulopathy (INR 1.6), lipid serum profile – nk	1 mo – family screening	12.0 nmol/mg protein/18 h (L); 17.2%	n.a.	n.a.	c.1177 T > G, p.Trp393Gly/c.1177 T > G, p.Trp393Gly	sinus node dysfunction and permanent pacemaker implantation at 1y; mild HS, thrombocytopenia (PLT 84), elevated ST (AST 1110, ALT 40), cholestasis (TB 12.3, DB 8.5), coagulopathy (INR 2.74), hypoalbuminemia, interstitial lung disease on chest X-ray, lipid serum profile – nk, cherry red spot, death due to heart failure	Died at 3 y
3	M Gypsy	3 y mild HS, elevated ST (AST 100, ALT 80), normal lipid serum profile, normal spirometry	2 mo – family screening	0 nmol/mg protein/18 h (L); 0%	41 (2 mo); 296 (5 y)	785 4364; 9.5 y	c.1177 T > G, p.Trp393Gly/c.1177 T > G, p.Trp393Gly	mild HS, elevated ST (AST 80, ALT 75), no cholestasis, normal coagulation profile, interstitial lung disease on chest X-ray, normal lipid serum profile, normal stature, normal neurologic examination, normal spirometry	9.5 y
4	F Gypsy	6 mo mild HS, elevated ST (AST 470, ALT 370), lipid serum profile: TC 186, LDL-C 104, HDL-C 9, TG 365)	6 mo – family screening	0.01 nmol/mg protein/18 h (L); 0.01%	488 (2.5 y)	26.8^#^ 25.6^#^; 2.5 y	c.1177 T > G, p.Trp393Gly/c.1177 T > G, p.Trp393Gly	mild HS, elevated ST (AST 170, ALT 150), no cholestasis, lipid serum profile: TC 264, LDL-C 205, HDL-C 18, TG 206, cherry red spot, normal stature, autism spectrum disorders	2.5 y
5	M Gypsy	6 mo mild HS, elevated ST (AST 160 ALT 130), lipid serum profile: TC 184, LDL-C 54, HDL-C 20, TG 548	6 mo – family screening	n.a.	n.a.	n.a.	c.1177 T > G, p.Trp393Gly/ c.1177 T > G, p.Trp393Gly	mild HS, elevated ST (AST 100, ALT 90), no cholestasis, normal lipid serum profile, cherry red spot, normal neurological examination	1 y
6	F Polish	2.5 y mild HS, elevated ST (AST 140, ALT 65), lipid serum profile: TC 298, LDL-C 224, HDL-C 14, TG 299	12 y	14 nmol/mg protein/18 h (L); 20%	1220 (12 y), 1600 (14 y)	274 13,670; 21 y	c.496G > A, p.Gly166Arg/ 3′ acceptor splice site mutation (g2610c)	moderate HS, elevated ST (AST 155, ALT 90), lipid serum profile: TC 290, LDL-C 229, HDL-C 18, TG 321, no cherry red spot, short stature, spirometry - nk	21 y
7	M Polish	1.5 y mild HS, normal ST, normal lipid serum profile	2 y	0.71 nmol/mg protein/18 h (L); 1%	3720 (2 y); 3300 (9 y); 3700 (14 y); 4190 (16 y)	n.a.	c.496G > A, p.Gly166Arg/ c.496G > A, p.Gly166Arg	moderate HS, normal ST, normal lipid serum profile, cherry red spot, interstitial lung disease on chest X-ray, hearing loss, normal stature, normal spirometry	18 y
8	M Polish	Splenectomy at 5 y, normal ST, normal lipid serum profile	12 y	22 nmol/mg protein/18 h (L); 31%	1 (12 y) Chitotriosidase deficiency - homozygote for a 24 bp duplication in the *CHIT1* gene	36 47; 18 y	c.866G > A, p.Arg289His/ c.496G > A, p.Gly166Arg	No hepatomegaly, normal ST, normal lipid serum profile, no cherry red spot, normal stature, spirometry - nk	18 y
9	M Polish	mild HS and thrombocytopenia (PLT 100–120) noted from years, lipid serum profile: TC 120, LDL-C 56, HDL-C 34, TG 155, normal spiormetry	50 y	112 pmol/spot/20 h (B); 56% (2nd measurement – 111 pmol/spot/20 h)	72 (50 y); 45 (54 y)	49 689; 54 y	c.107 T > C (p.V36A; rs1050228)/ c.107 T > C (p.V36A; rs1050228)	mild HS, thrombocytopenia (PLT 85), normal ST, lipid serum profile: TC 272, LDL-C 201, HDL-C 33, TG 191, no cherry red spot, normal spirometry	54 y
10	M Polish	Splenectomy at 48 y, normal ST, lipid serum profile: TC 140, LDL-C 82, HDL-C 27, TG 155, normal spirometry	49 y	11 nmol/mg protein/18 h (L), 15.7%	68 (49 y); 53 (51 y)	143 535; 60 y	c.940G > A, p.Val314Met/ c.940G > A, p.Val314Met	No hepatomegaly, normal ST, lipid serum profile: TC 112, LDL-C 41, HDL-C 39, TG 157,, no cherry red spot, normal spirometry	60 y
11	M Polish	1 y mild HS, normal PLT, lipid serum profile: TC 195, LDL-C 142, HDL-C 21, TG 164	45 y	1.5 ukat/kg protein (F); 5%	569 (45 y); 232 (60 y)	589 7035; 60 y	c.1400A > C, p.Tyr467Ser/ c.996delC, p.F333Sfs*52	mild HS, thrombocytopenia (PLT 110), normal ST, lipid serum profile: TC 256, LDL-C 197, HDL-C 18, TG 256, interstitial lung disease on chest X-ray and CT, deteriorating spirometry analyses: FEV1–3.44, VC max – 4.09, FEV1%VCmax – 84 (45 y), FEV1–3.37, VC max – 4.15, FEV1%VCmax – 78 (60 y), no cherry red spot,	60 y
12	F Polish	6 mo mild HS, normal ST, lipid serum profile: TC 140, LDL-C 98, HDL-C 18, TG 120	4 y	0.15 ukat/kg protein (F); 0.5%	908 (20 y)	529 5015; 40 y	c.1823_1825delGCC, p.delR610/ c.575delC, p.P192fsX62	mild HS, normal ST, cherry red spot, interstitial lung disease on chest X-ray and CT, deteriorating spirometry analyses lipid serum profile: TC 240, LDL-C 180, HDL-C 20, TG 220; deteriorating spirometry: FEV1–3.04, VC max – 3.09, FEV1%VCmax – 64 (20 y), FEV1–3.04, VC max – 3.11, FEV1%VCmax – 58 (40 y)	40 y
13	F Polish	10 y mild HS, normal ST, normal lipid serum profile	12 y	0 ukat/kg protein (F); 0%	171 (12 y); 200 (13 y)	n.a.	c.496G > A, p.Gly166Arg/c.496G > A, p.Gly166Arg	mild HS, normal ST, normal lipid serum profile, no cherry red spot, normal stature, spirometry - nk	17 y
14	F Polish	5 y massive HS, normal ST, normal lipid serum profile	14 y	0 ukat/kg protein (F); 0%	2140 (14 y)	n.a.	c.496G > A, p.Gly166Arg/c.496G > A, p.Gly166Arg	massive HS, normal ST, normal lipid serum profile, no cherry red spot, normal stature, spirometry - nk	21 y
15	M Polish	4 y mild HS, normal ST, normal lipid serum profile	10 y	n.a.	301 (10 y); 206 (15 y)	182 1256; 20 y	c.491G > T, p.Gly164Val/c.996delC, p.F333Sfs*52	mild HS, normal ST, normal lipid serum profile, no cherry red spot, normal stature, spirometry - nk	20 y
16	F Polish	mild S, normal ST, normal lipid serum profile	33 y	7 nmol/mg protein/18 h (L);10%	168 (33 y); 158 (34 y)	n.a.	c.996delC, p.F333Sfs*52/c.1142C > T, p.Ser381Phe	mild splenomegaly, normal ST, normal lipid serum profile, no cherry red spot, spirometry - nk	42 y

*M* Male; *F* Female; *SPC* Lysosphingomyelin; *HS* Hepatosplenomegaly; *n.a*. Not analyzed; *ST* Serum transaminases; *AST* Aspartate aminotransferase, *ALT* Alanine aminotransferase, *INR* International *TG* Triglycerides; *TC* Total cholesterol; *LDL-C* Low-density lipoprotein-cholesterol; *HDL-C* High-density lipoprotein-cholesterol; *nk* not known; *mo* Months; *y* years;

Reference values: ALT, < 18 months, < 55/60 U/L; 18 months–12 years, boys, < 40 U/L, girls, < 35 U/L; > 12 years, boys, < 26 U/L, girls, < 22 U/L; AST, < 52 U/L; total serum bilirubin, < 1.0 mg/dl; total cholesterol, < 170 mg/dl; LDL-C, < 110 mg/dl; HDL-C, > 45 mg/dl; TG, 1–9 years, < 75 mg/dl, 10–19 years, < 90 mg/dl; PLT, 150–450 K/μL

^*^Reference range for acid sphingomyelinase activity in leukocytes: 70-108 nmol/mg protein/18hr; skin fibroblasts 30 ± 10 ukat/kg protein; dried blood spot: 200-3500 pmol/spot/hr.

^**^Reference range for chitotriosidase activity in plasma: < 150 nmol/ml/hr.

^***^Reference range for SPC:4-55 nmol/l, SPC-509: 18-69 nmol/l.

^#^Reference range for SPC: 0.2-15, SPC-509: 0.15-3.7

## Results

### Patients - demographics

A total of 16 patients (9 males, 7 females) were enrolled into the study. 6 patients from one family were of Gypsy ancestry and the others were of Polish origin.

Twelve patients were diagnosed in childhood (age range: 1 year – 14 years) and 4 others in adulthood, the oldest one at the age of 50 years. The mean age at diagnosis was 20 years and the median was 12 years. 4 patients were diagnosed through family screening in early childhood.

### Presentation at diagnosis – Hepatosplenomegaly, serum transaminases and hematological parameters

Splenomegaly was noted in all the patients; two of them (Pt 8, 10) were splenectomized because of thrombocytopenia at the age of 5 and 48 years, respectively. The presence of lipid-laden macrophages in histopathological examination prompted the final ASMD diagnosis.

Hepatomegaly was observed in 14 (88%) out of 16 patients. All the patients had presented with a mild enlargement of the liver and spleen.

Elevated serum transaminases (several-fold above the upper limit of normal values) were noted in 6 (38%) out of 16 patients.

Apart from two splenectomized patients, one patient (Pt 9) had initially presented with thrombocytopenia.

Cholestatic jaundice was noted in 2 (13%) out of 16 patients. Those patients (Pt 1–2) also presented with coagulopathy (prolonged INR).

### Presentation at diagnosis – Biochemical analysis

Data on ASM activity were available for 14 out of 16 patients (9 in peripheral blood leukocytes, 4 in skin fibroblasts and 1 in dried blood spot). In case of 2 other patients the diagnosis was established only by molecular analysis of the *SMPD1* gene.

At the time of diagnosis, the chitotriosidase activity in plasma was available for 12 out of 16 patients. The mean level was 826 nmol/ml/hr. and it ranged from 40 to 3720 nmol/ml/hr. with the median at 236 nmol/ml/hr.; with the reference range up to 150 nmol/ml/hr. Chitotriosidase activity was strongly correlated with the volume of the liver and spleen. One patient (Pt 8) was diagnosed with chitotriosidase deficiency – homozygote for the 24 bp-duplication in the *CHIT1* gene.

### Molecular analysis

Eleven various *SMPD1* gene variants were identified in the study with 10 of them previously described in the literature. In one patient (Pt 15) we found a previously unreported heterozygous variant in exon 2 (c.491G > T, p.G164 V) which is located in highly conserved nucleotides and moderately conserved amino acids positions with moderate physicochemical differences between the amino acids glycine and valine.

Missense mutations were the most common types of genetic lesions, comprising 75% of all alleles. The most common mutation was p.G166R comprising 25% of alleles. It was found in homozygosity in three patients and in heterozygosity in three others. This cohort contained only one patient, a heterozygote for p.delR610 mutation. One patient (Pt 12) was identified as a homozygote for p.V36A variant, which was referred to as a non-synonymous single nucleotide polymorphism by Rhein et al. [13].

### Follow-up

The mean time of follow-up in our study was approximately 10 years (range: 6 months – 36 years).

### Follow-up – Hepatosplenomegaly, serum transaminases, hematological parameters and biochemical analysis

The liver and spleen volume was stable during the follow-up.

Elevated serum transaminases observed in 6 patients at diagnosis remained comparable during the follow-up. Rising serum bilirubin levels and deterioration in liver synthetic function – hypoalbuminemia, coagulopathy (prolonged INR), were observed in 2 patients (Pt 1–2) who, at the time of diagnosis, presented with hepatosplenomegaly and cholestasis. They died at the age of 1.5 and 3 years, respectively, due to problems not related to liver disease (see Table 1).

Two patients who had undergone splenectomy were excluded from the analysis of the hematological parameters, because the abnormalities were related to hypersplenism. Over time, platelets tended to decrease in 3 patients and remained stable in other patients. At the last follow-up, thrombocytopenia was noted in 4 (28%) out of 14 patients.

At the last follow-up (years 2017–18), the result of chitotriosidase activity in plasma was available for 9 patients, it decreased in 5 of them and slowly increased in 4 others. SPC and SPC-509 levels in plasma were assessed in 9 out of 16 patients at the last follow-up. In comparison to controls, SPC was elevated in 7 (78%) patients, while in 2 patients only a slight elevation (Pt 10, 15) was observed. SPC-509 was elevated in 8 (89%) out of 9 patients.

### Cholesterol and cardiovascular assessment at diagnosis and follow-up

Data on lipid serum profile were available in 14 out of 16 patients at the time of diagnosis. They were completely normal in 7 (50%) patients. In 7 (50%) other persons lipid serum profile abnormalities were defined as follows: elevated TC (6/7), elevated LDL-C (3/7), elevated TG (7/7), decreased HDL-C (7/7).

A the last follow-up visit, data on lipid serum profile were completely normal in 8 patients – they had normalized in 1 patient (Pt 5). In 6 other patients, lipid serum profile abnormalities were defined as follows: elevated TC (5/6), elevated LDL-C (5/6), elevated TG (6/6), decreased HDL-C (6/6).

### Pulmonary assessment at diagnosis and follow-up

Chest radiography and CT-scanning were used to diagnose interstitial lung disease in 5 (31%) patients out of the whole cohort. Data on pulmonary function tests (spirometry analyses) were available in all of them with a mild obstructive disease found in 2 out of 5 patients (Pt 11–12). During the follow-up, an insignificant deterioration of these parameters was observed.

DLCO parameters were not analysed in the study.

### Neurological assessment and ophthalmological findings

Cherry-red spots were reported in 5 Gypsy children from one family (Pt 1–5) and 1 Polish adult patient (Pt 12). All of them presented with the absence of neurological impairment during long-term follow-up.

### Growth parameters

Only 1 (13%) out of 8 children in the study had a short stature which was assigned as a familial short stature.

## Discussion

In the study we described genotypic and phenotypic characteristics of 16 patients from Poland with chronic visceral ASMD. Long-term follow-up results were characterised of this group of patients.

ASMD has a predominant expression in the liver and spleen due to the accumulation of sphingomyelin and other lipids in the macrophage-monocyte system and also hepatocytes [12, 14, 15]. All our patients had initially presented with splenomegaly and almost all of them with hepatomegaly. In some of the patients, the liver and spleen enlargement were diagnosed incidentally and had constituted the sole signs presenting for many years before the final ASMD diagnosis. Similarly, in the literature, hepatosplenomegaly is the most commonly reported clinical finding at the initial presentation of the disease [5–9].

Thus, an elevated serum chitotriosidase activity, which is specifically expressed by activated macrophages, could be noted [16–19].

Liver fibrosis and finally cirrhosis occur in the natural course of ASMD [5, 14, 15]. Therefore, elevated serum transaminases may be noted. On the basis of our observations, it is worth emphasizing that only a mild to moderate level of elevation is reported, reaching several-fold above the upper limit of normal values. Based on the obtained data, chronic visceral ASMD may also be a reason for impaired liver synthetic function. Serum bilirubin concentrations are commonly normal, except for children who could present with cholestatic liver disease [5].

The natural course of liver disease is usually non-progressive and it is comparable with data from other reports [6–9, 14–16, 20]. However, a subset of patients is described in the literature, who are at an increased risk for hepatic failure occurrence [21, 22]. Five Gypsy patients (Pt 1–5) from one family, affected with p.T393G missense mutation in a homozygous state, had presented with a variable disease course. Two of them (Pt 1–2) had cholestatic liver disease with coagulopathy which had progressed to chronic liver failure but they had finally succumbed due to reasons not related to liver disease (*Neisseria meningitidis* sepsis and heart failure, respectively). In 3 other patients the disease course was mild to moderate with stable liver disease. It shows that, apart from the genotype, other factors such as environmental ones may also contribute to disease severity – patients 3–5 were under family foster care. Furthermore, there is 1 more child in this family, currently aged 5, who is healthy (normal ASM activity).

Several cases of adult ASMD patients with cirrhosis and portal hypertension were reported in the literature [16, 22, 23]. In our cohort, apart from 2 splenectomized patients, 3 other patients presented with signs of portal hypertension in the form of a mild thrombocytopenia (a feature of hypersplenism).

Macular cherry-red spots constitute the ophthalmologic finding caused by the accumulation of sphingomyelin in the retina. They were reported in the literature, both in neuronopathic and non-neuronopathic forms of ASMD [3–9, 24]. In our cohort, cherry-red spots were found in five Gypsy children from one family (Pt 1–5) and also in 1 adult Polish patient (Pt 10). None of the patients with macular cherry-red spots had presented with signs or symptoms of central nervous system involvement. Notably, some patients with chronic visceral ASMD could present with macular cherry-red spots.

Lipid abnormalities are characteristic for ASMD featured with an abnormal lipid profile in the serum showing increased levels of serum TC, LDL-C, TG and decreased levels of HDL-C [1, 2, 6–9, 20, 24–26]. The finding of dyslipidemia was reported in half of the patients from this cohort. The most frequent features included elevated TG levels and decreased HDL-C levels. These abnormalities might be associated with the development of atherosclerotic heart disease. Contrary to other reports in the literature, none of our adult patients presented with symptomatic atherosclerotic heart disease [2, 25].

About one third of patients in this study have a diagnosis of interstitial lung disease. The pathophysiology of the lung disease is associated with the infiltration of the alveolar septa, bronchial walls, and pleura by lipid-laden macrophages potentially leading to a progressively worsening restrictive pattern on pulmonary function testing [6–9, 27, 28]. Only slow and non-significant progression of pulmonary disease observed on the basis of pulmonary function tests was reported in our study group emphasizing controlled lung involvement in chronic visceral ASMD.

To date, about 200 pathogenic variants in the *SMPD1* gene have been reported. *SMPD1* gene mutations profile described in our study was similar to others in the literature where missense mutations constituted the most commonly reported ones [13, 29–33].

The most common variant in the study was a missense mutation p.G166R and it was found in 6 patients; in 3 of them in a homozygous state and in 3 others in a heterozygous state. Those patients were living in the same region, so we could hypothesize on a slight founder effect.

The molecular variant p.F333Sfs*52, which was found in heterozygosity in 2 patients (Pt 15, 16) constitutes a common Ashkenazi Jewish type A mutation and is related to a severe phenotype of the disease [13, 29]. Based on our study, we could hypothesize about a protective character of the second mutation, p.G164 V and p.S381P, respectively, responsible for a mild phenotype of chronic visceral ASMD.

To better characterize the disease progress and genotype-phenotype correlation, we used two serum biomarkers, the deacylated form of sphingomyelin – lysosphingomyelin (SPC) and SPC analog – lysosphingomyelin-509 (SPC-509). 9 patients from the study group underwent SPC and SPC-509 measurement at the last follow-up visit. It was a single measurement as these biomarkers had the levels rising with time.

SPC was found to be a promising biomarker in ASMD and SPC-509 in both ASMD and Niemann-Pick disease type C [34–37]. In 2 study patients (Pt 8 and 9) SPC levels were found in a control range, so we could categorize them as a very mild phenotype. In Patient 8, the second biomarker SCP-509, was also found in a control range. This patient was splenectomized in infancy, which put a new light on those two biomarkers suggesting that splenectomy may affect SPC and SCP-509 levels. Based on those observations, we propose to measure ASM activity in patients with normal SPC-509 who had undergone splenectomy.

Patient 9 was also found to have elevated SPC-509 levels and to be chitotriosidase-deficient. This patient was identified as a homozygote for a known SNP, p.V36A. The fact about normal SPC levels and elevated SPC-509 levels, suggests that the elevation of SPC509 only, should not exclude ASMD diagnosis in patients presenting with a mild phenotype. In such cases, we propose to measure ASM activity prior to *SMPD1* gene mutation analyses.

In 1 patient with an elevated SPC (Pt 10), there was only a mild level of elevation. This patient was found to be a homozygote for p.G314Val mutation presenting with no history of disease up to adulthood, when he had undergone splenectomy. Based on the serum biomarkers levels and the long-term follow-up of this patient, we could correlate p.G314Val variant with a mild phenotype of ASMD.

## Conclusions

In the group of chronic visceral ASM deficient patients, spleen enlargement was always present. Liver enlargement was observed in a majority of patients and was associated with moderately elevated serum transaminases.

Macular cherry-red spots were also noted in chronic visceral ASM deficient patients.

All the patients, apart from one (with familial short stature), presented with normal stature.

The combined measurement of lysosphingomyelin (SPC) and lysospingomyelin-509 (SPC-509) was a highly essential method for the assessment of ASMD course. Serum chitotriosidase activity was mostly conclusive in cases with clinically significant hepatosplenomegaly.

Chronic visceral ASMD could have a mild course with a relatively good outcome.

Other factors such as environmental ones could contribute to the disease severity.

## Abbreviations

ASMD: acid sphingomyelinase deficiency
CT: computed tomography
HDL-C: high-density lipoprotein-cholesterol
LDL-C: low-density lipoprotein-cholesterol
SMPD1 gene: sphingomyelin phosphodiesterase 1 gene
SPC: lysosphingomyelin
SPC-509: lysosphingomyelin-509
TC: total serum cholesterol
TG: triglycerides (TG)
